# The unfairness we feel: How positive and aggressive affect could shape relative deprivation and aggression

**DOI:** 10.1186/s40359-025-02732-x

**Published:** 2025-04-17

**Authors:** Yara J. Kassab, Georg Halbeisen, Eva Walther

**Affiliations:** 1 Department of Psychology, University of Trier, Trier, Germany; 2 University Clinic for Psychosomatic Medicine and Psychotherapy, Medical Faculty, Campus East-Westphalia, Ruhr-University Bochum, Virchowstr 65, Luebbecke, 32312 Germany

**Keywords:** Personal relative deprivation, Interpersonal aggression, Game task, Political psychology, Aggressive affect

## Abstract

**Background:**

Relative deprivation (RD), the belief of being unfairly disadvantaged compared to a standard, has frequently been linked to aggressive behaviors. This study explored how affective experiences are associated with the perception of unfairness (i.e., RD) and, thus, influence aggressive behavior indirectly.

**Methods:**

*N* = 184 participants were randomly assigned to one of two conditions of a game task, in which they experienced either deprivation or no deprivation as the difference between own rewards and the rewards of a fictitious other player. We assessed the subjective perception of RD and affective experiences attributed to the game before measuring aggressive behavior towards the other player in a point subtraction task.

**Results:**

Sequential mediation analysis suggested that increases in aggressive affect and decreases in positive affect could be linked to perceiving the game as more unfair for deprived participants, which in turn increased rates of aggressive behavior.

**Conclusions:**

Adding to the existing literature, these findings suggest that RD could not only lead to aggression through an affective route but that affective experiences potentially alter perceptions of RD and thus aggressive behavior indirectly.

**Supplementary Information:**

The online version contains supplementary material available at 10.1186/s40359-025-02732-x.

## Background

Relative deprivation (RD), the subjective perception of being unfairly disadvantaged compared to a standard [33], has been linked to a variety of individual and social consequences, such as aggressive behavior (e.g., [17]), crime (e.g., [32, 38]), or even addiction (e.g., [3]). According to the theoretical and meta-analytic review by Smith et al. [31], RD leads to these consequences through several steps. First, a social comparison must take place by an individual that, second, leads to the perception that the individual is disadvantaged, that is, relatively deprived. Third, the subjectively perceived disadvantage must be viewed as unfair, which could lead to feelings of hostility and anger and thus fuel aggressive behavior. Indeed, due to their close association with aggressive behavior, feelings of hostility and anger are often referred to as “aggressive affect” in aggression research [1], p. 2) in contrast to other negative states (e.g., sadness or embarrassment; [39]). Thus, RD presumably leads to aggression through an affective route. At the same time, however, affective experiences may recursively alter perceptions of unfairness itself [28], suggesting that aggressive and other affective experience could be linked to modified perceptions of RD. To further our understanding of the relation between the subjective perception of RD and aggressive behavior, we aim to explore how positive and aggressive affect linked to a social comparison are associated with the perception of unfairness and, thus, aggressive behavior indirectly.

### Affective routes to subjective RD

Exploring the relations between unfavorable social comparisons, affective experiences, and social behaviors has been the ongoing purpose of RD research since Stouffer introduced the concept to explain the frustration of the U.S. Army Air corpsmen over promotions [33]. The initial research did not assess RD directly and thus did not introduce a prototypical measure but instead inferred RD as an explanation for the collected data,since then, however, several measures have been developed to assess RD´s cognitive (the perception of a difference in treatment, income, or gratification) and affective (feelings of anger and resentment) derivates and their interactions [31]. Crosby [6] argued in his model of RD that people feel resentment about failure to possess a desired something only when they see that similar others possess it, feel entitled to possess it, and think that its possession is feasible without blaming themselves for the failure of not possessing it. The disadvantage to others must thus be viewed as undeserved to lead to the subjective experience of RD, and this perception may invoke aggressive affect, i.e., feelings of anger and resentment [25, 30]. Indeed, Smith and colleagues [31] pointed out that the subjective experience of RD more than the objective disadvantage affects people´s aggressive affect and behaviors and therefore advised to measure subjective rather than objective deprivation.

Consistent with the original conceptualization, studies that explored the link between RD and aggression focused on aggressive affect as the consequence of subjective RD and as a mediator of aggressive behaviors. For example, a recent longitudinal investigation among Chinese university students found that experiences of and rumination about anger mediated the link between subjective RD and aggressive behavior across five waves of data [4]. Similarly, in studies that manipulated RD and measured affective states [15, 16], RD led to aggressive behavior via increased perceptions of disadvantage and, subsequently, hostile feelings. It is important to note, however, that previous studies rarely assessed positive affect following manipulations of RD, leaving room for interpretation whether, for instance, reduced positivity could similarly increase aggressive behaviors.

Previous work has also not explored a complementary pathway in which aggressive affect links to increased aggressive behavior through a recursive association to perceptions of RD. Based on the ideas of recursive processes in appraisal theories of emotion [28] and of bi-directional influences between cognitions, affect, and arousal in the general aggression model [1], we speculated that affective experiences may not only follow from but could also inform perceptions of RD, for example, by anger directing attention to “unfair” aspects of the situation or evoking memories of previous unfair treatments (e.g., [11, 12, 29]). Indeed, Cohen et al. [5] found that angry people pay more attention to anger-related stimuli than to similar neutral stimuli. Furthermore, [2] found that angry participants judged the behavior of others as more intentional and a perpetrator as having more causal control and more willing to punish the wrongdoer than participants in a neutral state. Moreover, because positive affective reactions have been found to bias attention toward positively valued stimuli [34], the reduction of positive affect could make own advantages more difficult to detect. We may therefore expect that increased aggressive affect and reduced positive affect would be linked to perceiving relative disadvantages as more unfair, thus increasing perceptions of RD. Longitudinal survey data indeed supports a cross-lagged effect of anger increasing later perceptions of RD [4, 35]. To the best of our knowledge, these links, however have not been previously explored in experiments that manipulated RD, which could give a clearer indication of whether heightened aggressive affect—and reduced positive affect—are linked to increased perceptions of RD beyond those levels expected based on objective disadvantages.

### The present research

Using a recently validated RD game [19], the present study explored the interrelations between aggressive and positive affect, subjective RD, and aggressive behavior. Similar to other experimental games with meaningful choices [36], participants placed bets and directly experienced objective unfairness over several rounds as they observed another player receive more rewards for placing similar bets (RD condition), compared to a neutral control condition in which rewards are distributed fairly. Afterwards, we measured the subjective perception of RD and positive and aggressive affect linked to the game, and participants’ aggressive behavior in a Point Subtraction Aggression Paradigm (PSAP; [13]) that offered the opportunity to destroy the other player’s coins (the rate of destruction served as the measure of behavioral aggression. For exploration, we also measured traits that could moderate the effect of deprivation, i.e., Social Dominance Orientation [37] and Disgust Propensity [8], see Supplementary Material). We previously found participants in the RD condition to attribute more aggressive and less positive affect to the RD game [19], but we did not investigate whether and how these experiences are linked recursively to the perception of RD or aggressive behavior. In the present study, we expected to replicate the RD game’s effects on subjective RD, affective experiences, and aggressive behavior, and we speculated that increased aggressive affect and reduced positive affect would be associated with more pronounced perceptions of RD and thus aggressive behavior indirectly.

## Methods

### Participants and design

*N* = 184 undergraduate university students (75 women, 96 men, 13 non-specified, *M*_age_ = 22.16, age range: 18 – 31 years) participated in exchange for course credit and money (up to 3.70€) across two experiments with a random assignment to a 1 × 2 (RD-manipulation: RD vs. no-RD) between-participants design. The studies originally intended to investigate the effects of a minimal group manipulation on RD and aggression, and thus contained an additional group context factor (for example, the other player was designated as studying a different major, or at another university, see Supplementary Material). However, because the minimal group conditions exerted no effect on either RD or aggression (see Supplementary Materials), and because the procedures were identical except for the designation of the other player, we combined both datasets to increase the power for the present analysis. Key explanatory variables (reward differences, subjective RD, positive and aggressive affect, see below) did not differ between datasets, all *p*s > 0.21. However, because the overall rates of aggression differed, *p* < 0.001, we included dataset as a covariate in the analysis (although inferential patterns were unaffected by the covariate’s inclusion, see below). Based on the effect of RD conditions on aggression in Kassab et al. [19], *η*^*2*^ = 0.12, the combined sample had a power of *1-β* = 0.99 to detect a main effect of RD condition at *α* = 0.05 [9]. All data analyses were conducted after data collection was terminated. The raw data supporting the conclusions of this article will be made available by the authors, without undue reservation.

### Measures and procedure

The research was conducted in a manner consistent with the APA’s ethical principles in the conduct of research with human participants. All participants gave informed written consent. The studies were not pre-registered. Participants were recruited via the university’s participant management system. The experiments were conducted in a laboratory equipped with four PCs separated by partition walls. After providing informed consent, participants were informed the experiment consisted of a computer game, in which they had the opportunity to win cash money by taking turns with another player (Player 2). The instructions stated that the experiment was entirely operated by the computer, and there were no further interactions between the experimenter and the participant.

Starting the task, participants indicated their study major or university membership (for the previously mentioned reasons), and completed a Social Dominance Orientation (SDO) short version questionnaire [37], after which they were introduced to the RD game developed and validated by Kassab et al. [19]. Participants learnt they were to take turns of placing bets with Player 2 to win real money, represented by virtual coins. It was explained that: “the money you can earn will be disbursed at the end of the game. The game consists of a number of trials. On each trial you are asked to press a key in the center of the screen showing the values 1 to 10. You can either win or not win the number of coins as indicated by the key value. Your obtained coins will be displayed in your coin-box on the screen. Every coin is worth 0.05 Euros.” Players were taking turns and observed each other’s bets and winnings. Each player made a total of 20 bets. Unbeknownst to the participants, the game incorporated an algorithm that adjusted the winning chances of the participant and Player 2 in a way that gradually led to unequal outcomes in the RD condition and similar outcomes in the no-RD condition [19]. At the start of the game, the winning chances for receiving an outcome were identical for both the participant and Player 2 such that the chance of winning one coin by choosing key 1 was 95% and it decreased by 10% with every increase in key value. The choices of Player 2 always varied between ± 2 of the chosen value of the participant, assuring similar outcomes for both players (to enhance the game’s credibility, decision times for Player 2 also varied between ± 20% of the decision time of the participant). While the winning chances did not change throughout the game in the no-RD condition, the winning chances in the RD condition remained identical for both players only in the first block of 50% of trials. After this first block of fair trials, which was realized to increase the contrast to the subsequent unfair trials, the winning chances in the RD condition for the participant decreased proportionally with every further trial and increased for Player 2. This way, the algorithm ensured that participants in the RD condition ultimately won fewer coins than Player 2. The difference in the amount of won coins served as the manipulation check.

Following the game, the dependent variables were assessed. Participants completed the six-item personal perception of relative deprivation scale (PPRD, Cronbach’s α = 0.90; [19]) as well as items assessing positive affect and aggressive affect attributed to the RD. Specifically, on a 7-point scale, ranging from 1 (*not at all*) to 7 (*very much*), participants rated how happy, satisfied, and pleased (Cronbach’s α = 0.79), as well as how angry, resentful, and distressed (Cronbach’s α = 0.89) they felt during the game. We chose to measure rather than manipulate affect, given that, for example, an induction of anger would have likely interfered with risk-taking behavior and thus outcomes in the RD-game [10]. Furthermore, because disgust has been shown to affect aggressive behavior [26], we also assessed participants’ moral- (5 items) and pathogen-disgust (6 items) on a five-point scale [8] as a potential moderators. However, although disgust propensity was related to aggressive behavior and pathogen-disgust buffered against aggression (as shown previously, [26]), including either disgust propensity or SDO into the mediation analyses did not change the findings, and these results are therefore not further reported here (but see Supplementary Material).

As in Kassab et al. [19], aggressive behavior against the other player following the RD-game was assessed using the Point Subtraction Aggression Paradigm (PSAP [13]). Participants had a predefined amount of time to destroy the other player’s rewards by clicking on a coin until it virtually exploded (4 clicks were needed). Because the number of coins won by Player 2 varied based on the conditions’ distribution algorithm and the participants preferences for betting on higher vs. lower winnings, the amount of time was based on Player 2’s relative advantage (0.5 s per coin advantage + 20 s), ensuring that the instrumental value of aggressive behavior (i.e., the effect on Player 2’s advantage) remained comparable between conditions. Each click, destroyed coin, and the available time were registered, and the rate of destroyed coins per second (ensuring comparability between conditions and participants) was analyzed as a measure of aggression. The procedures concluded after demographic and additional exploratory assessment (not further reported). They were then thanked, debriefed, and awarded the money they won in the game.

### Analysis

Differences in rewards between the participant and Player 2, the subjective perception of RD (PPRD mean scores), and the rate of destroyed coins in the PSAP were submitted to separate 1 × 2 (RD-manipulation) between-participants ANCOVAs, with sample origin as covariate. Means for positive and aggressive affect were submitted to a 2 (RD-manipulation) × 2 (affect: aggressive vs. positive) ANCOVA, respectively. ANOVAs without the covariate produced nearly identical results (see Table 1). We explored the interrelations between aggressive affect, subjective RD, and aggressive behavior with sequential mediation analysis, using RD as predictor *X*, positive affect, aggressive affect and PPRD as mediators *M*_*1,*_* M*_*2*_ and *M*_*3*_, respectively, and number of click as criterion *Y* (again, sample origin was included as covariate).

**Table 1:** Results of unadjusted comparisons (ANOVA)

DV	Factor	SS	df	MS	F	p	η^2^_p_
Reward Diff	Intercept	268,977	1	268,977	1472.7	< .001	.89
	RD	268,825	1	268,825	1471.8	< .001	.89
	Error	33,242	182	183			
PPRD	Intercept	2846	1	2846	2687.7	< .001	.94
	RD	268	1	268	253.3	< .001	.58
	Error	193	182	1			
Affect. Exp	Intercept	3712	1	3712	3258.7	< .001	.95
	RD	4	1	4	3.6	.06	.02
	Error(RD)	207	182	1			
	affect	52	1	52	23.8	< .001	.12
	RD * affect	122	1	122	55.8	< .001	.24
	Error(affect)	397	182	2			
Aggression	Intercept	7.90	1	7.90	81.8	< .001	.31
	RD	0.36	1	0.36	3.7	.05	.02
	Error	17.59	182	0.10			

*DV* dependent variable, *SS* Square sums, *df* degrees of freedom, *MS* mean square, *RD* relative deprivation manipualtion, *PPRD* personal perception of relative deprivation

The significance level for all analyses was set at *p* ≤ 0.05. Post hoc pairwise comparisons report Bonferroni-adjusted *p*-values for multiple comparisons. Effect sizes are reported as *η*_*p*_^*2*^. Variable values are reported as means and standard deviations (SDs). The data were aggregated and analyzed with IBM SPSS 28 (IBM Corp., 2021). We used PROCESS v4.2 [18] with boot-strapped (10,000 samples) percentile bootstrap 95% confidence interval (CI) for mediation analysis (model 80).

## Results

### Reward differences

The algorithm produced the intended disadvantages in the RD conditions. The 1 × 2 (RD-manipulation) between-participants ANCOVA on reward differences between the participant and the Player 2 showed that Player 2 received more coins in the RD compared to the no-RD condition (*M*_*RD*_ = 76.5, *SD* = 17.4 vs. *M*_*noRD*_ = 0.1, *SD* = 8.0), *F*(1, 181) = 1463, *p* < 0.001, η^2^_p_ = 0.89.

### Subjective RD

The 1 × 2 (RD-manipulation) ANCOVA on PPRD scores showed the RD-game succeeded to be personally perceived as unfair, *F*(1, 181) = 252.17, *p* < 0.001, η^2^_p_ = 0.58, with participants in the RD condition reporting higher levels of perceived deprivation as compared to participants in the no-RD condition (*M*_*RD*_ = 5.14, *SD* = 1.15 vs. *M*_*noRD*_ = 2.73, *SD* = 0.89).

### Affective experiences attributed to the RD game

The 2 (RD-manipulation) × 2 (affect: aggressive vs. positive) ANCOVA, with affect as repeated-measures factor, showed a main effect affect, *F*(1, 181) = 5.92, *p* = 0.02, η^2^_p_ = 0.03, qualified by the RD-manipulation × affect interaction, *F*(1, 181) = 55.88, *p* < 0.001, *η*^*2*^ = 0.24. Pairwise comparisons revealed that participants reported significantly less positive and more aggressive affect in the RD condition than in the no-RD (*M*_*RD*_ = 2.87, *SD* = 1.08 vs. *M*_*noRD*_ = 4.23, *SD* = 1.09 and *M*_*RD*_ = 3.27, *SD* = 1.65 vs. *M*_*noRD*_ = 2.33, *SD* = 1.26, respectively), *p*s < 0.001; other effects, all *F*s < 3.6, all *p*s > 0.06.

### Aggressive behavior

Effects on aggressive behavior, i.e., the rate of destroyed coins of Player 2, were analyzed in a 1 × 2 (RD-manipulation) between-participants ANCOVA, which revealed the expected main effect of the RD-manipulation, *F*(1, 181) = 4.01, *p* < 0.05, η^2^_p_ = 0.02. There were higher rates of aggressive behavior in the RD as compared to the no-RD condition (*M*_*RD*_ = 0.25 coins/sec, *SD* = 0.33 vs. *M*_*noRD*_ = 0.16 coins/sec, *SD* = 0.29).

### Sequential mediation analysis

**Fig. 1:**
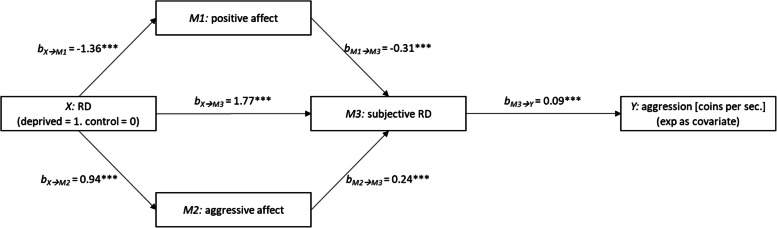
Mediation model with RD condition as predictor X (coded 1 0 for RD and no-RD conditions, respectively), positive affect, aggressive affect and PPRD as mediators M1, M2 and M3, and aggression (coins destroyed per second) as criterion Y (with sample cohort as covariate)

We explored whether positive and aggressive affect muted or intensified RD and/or directly linked to aggression when deprived in mediation analysis. The sequential mediation model with RD condition as predictor *X* (coded 1 0 for RD and no-RD conditions, respectively), positive affect, aggressive affect and PPRD as mediators *M*_*1,*_* M*_*2*_ and *M*_*3*_, and aggression as criterion *Y* (with sample cohort as covariate) is displayed in Fig. 1. Consistent with the previous analysis, RD condition predicted reduced positive affect, *b*_*X➔*__*M1*_ = -1.36, *p* < 0.001, and increased aggressive affect, *b*_*X➔*__*M2*_ = 0.94, *p* < 0.001. PPRD was predicted by RD conditions, *b*_*X➔*__*M3*_ = 1.76, *p* < 0.001, lowered positive affect, *b*_*M1➔*__*M3*_ = -0.31, *p* < 0.001, and higher aggressive affect, *b*_*M1➔*__*M3*_ = 0.24, *p* < 0.001. PPRD, in turn, predicted aggression, *b*_*M➔3*__*Y*_ = 0.09, *p* < 0.001. Aggressive and positive affect did not predict changes in aggression,* b*_*M➔1*__*Y*_ = 0.02, *p* = 0.46 and *b*_*M➔2*__*Y*_ = 0.03, *p* = 0.12, respectively. The boot-strapped model confirmed the presence of three indirect effects: An indirect effect for the RD → PPRD → aggression path, *IE* = 0.16, 95% CI [0.08; 0.25], an indirect effect for the sequential RD → positive affect → PPRD → aggression path, *IE* = 0.04, 95% CI [0.02; 0.06], and an indirect effect for the sequential RD → aggressive affect → PPRD → aggression path, *IE* = 0.02, 95% CI [0.01; 0.04]. There was a negative direct effect (DE) of condition on aggression when controlling for the influence of the mediators, DE = -0.13, 95% [-0.26; -0.01], and no simple mediations via positive affect, IE = -0.02, 95% [-0.07; 0.03], or aggressive affect, IE = 0.02, 95% [-0.01; 0.05].

In order to tentatively evaluate the degree to which the data support the hypothesized mediation model, we estimated alternative mediation models that presuppose different causal pathways. Specifically, we evaluated a reversed sequential mediation model in which positive and aggressive affect mediate the effect of PPRD on aggression (model 81) as well as a simple parallel mediation model with positive affect, aggressive affect, and PPRD as mediators of the link between RD and aggression (model 4). In both analyses, only the RD → PPRD → aggression paths were significant (see Table 2). Although it is sometimes argued that this result could suggest the data are more compatible with the hypothesized vs. alternative models (e.g., [21]), it is important to note that all tested models belong to the same equivalence class and thus it cannot be guaranteed which model is ‘true’ [27].

**Table 2:** Direct and indirect effects for affect-first (Model 80) and PPRD-first (Model 81) sequential mediation model. Parallel simple mediation (Model 4) for comparison

Model	Effect	Estimate	SE	LLCI	ULCI
80	Direct (RD → aggression)	-0.13	0.07	-0.26	-0.01
	TOTAL Indirect	0.22	0.05	0.13	0.32
	RD → Pos.aff. → aggression	-0.02	0.03	-0.08	0.03
	RD → Aggr.aff. → aggression	0.02	0.02	0.00	0.06
	RD → PPRD → aggression	0.16	0.05	0.08	0.26
	RD → Pos.aff. → PPRD → aggression	0.04	0.01	0.02	0.07
	RD → Aggr.aff. → PPRD → aggression	0.02	0.01	0.01	0.04
81	Direct (RD → aggression)	-0.13	0.07	-0.26	-0.01
	TOTAL Indirect	0.22	0.05	0.13	0.32
	RD → PPRD → aggression	0.22	0.06	0.11	0.34
	RD → Pos.aff. → aggression	0.00	0.01	-0.02	0.01
	RD → Aggr.aff. → aggression	-0.02	0.01	-0.05	0.00
	RD → PPRD → Pos.aff. → aggression	-0.02	0.02	-0.06	0.03
	RD → PPRD → Aggr.aff. → aggression	0.04	0.03	-0.01	0.09
4	Direct (RD → aggression)	-0.13	0.07	-0.26	-0.01
	TOTAL Indirect	0.22	0.05	0.12	0.32
	RD → PPRD → aggression	0.22	0.06	0.10	0.34
	RD → Pos.aff. → aggression	-0.02	0.03	-0.08	0.03
	RD → Aggr.aff. → aggression	0.02	0.02	-0.01	0.06

*RD* relative deprivation manipualtion, *PPRD* personal perception of relative deprivation, *Aggr.aff* aggressive affect, *Pos.aff.* positive affect

## Discussion

Inequalities are a major risk factor for conflict. From late antiquity (Aristotle 350 B.C./1984) to frustration-aggression theory [7], the idea exists that dissatisfaction is driven by inequality. However, there is also consensus that it is not objective inequality but the subjective experience of RD resulting from social comparison that leads to aggressive behavior [22]. The present study explored the mechanism underlying the RD-aggressive behavior link. We speculated that experimentally-induced RD and therefore the state of a disadvantaged comparison reduces positive and promotes aggressive affect that could mute or intensify the perception of being unfairly treated, which in turn increases aggressive behavior.

First, the findings replicated previous results, showing that experimentally induced RD promotes aggression and that participants report significantly less positive and more aggressive affect in the RD compared to no-RD condition [19]. In the RD condition, more coins of the other player than in the control condition were destroyed. Second, and essential for the present work, we explored whether increased aggressive affect and reduced positive affect would be linked to pronounced subjective perceptions of unfairness and/or directly linked to aggression when deprived in a mediation analysis. Here, we found three indirect effects: an RD → PPRD → aggression path, and two separate RD → affect → PPRD → aggression paths, for both positive and aggressive affect. In line with our speculation, perceiving oneself as being unfairly treated may not only follow by a disadvantaged state compared to others but could be linked to an increase of aggressive affect as well as the decline of positivity. Thus, they hint at a possible role of positive and aggressive affect in shaping the perception of unfairness and thus aggressive behavior indirectly.

Notably, we did not find that the affective experience reported by participants linked the perception of RD to aggression in additional mediation analyses (see above), as previously found in other studies that manipulated RD and measured affective states [15, 16]. It is important to acknowledge that previous studies did not test for the specific associations we obtained, and that there is no logical contradiction between assuming that, for example, aggressive affect mediates between perceptions of unfairness and aggressive behavior, and that aggressive affect mediates between objective unfairness and perceiving the situation as unfair. In fact, this is the idea of recursive processes in appraisal theories of emotion [28] and of bi-directional influences between cognitions, affect, and arousal in the general aggression model [1]. However, it is also important to discuss why past and present findings might have diverged.

First, there are some systematic differences in the operationalization of RD and affective experiences which may account for the different mediation patterns (see [15, 16]). Participants in our study were repeatedly deprived during the game task, whereas previous studies relied on false-feedback procedures to inform participants of their relative disadvantage only once. The repetitive nature of experienced discrimination in our game task may have created opportunities for affective experiences to aggravate perceptions of unfairness, allowing us to detect a mediation pattern in which affect preceded perceptions of RD. Second, at the same time, our assessment of affect was more contextualized than in other studies (e.g., [15, 16]), focusing on affective experiences directly experienced during the game in contrast to the previously used global assessments of state hostility (i.e., feeling angry *in general*). While a contextualized measure provides advantages in terms of detecting an effect of the RD manipulation, a general measure might be more reliant on information relevant to a behavioral decision (e.g., a lingering aggressive state) and thus more prone to emerge as a mediator of aggression [14]. Third, we must also acknowledge further incidental differences. For example, whereas we recruited mostly undergraduate students to participate in laboratory studies, [15, 16] recruited more diverse samples using crowdsourcing platforms. Study settings and sample characteristics can influence scale variances (e.g., [20]) and thus also the results of mediation analyses. Moreover, people in our study received rewards contingent on their behavior in the game rather than for merely participating, which could lead to differences in engagement and the quality of affective experiences. Without extensive re-analyses of these datasets, we can only speculate about the diverging patterns retrospectively.

The diverging findings notwithstanding, the question of when relative deprivation leads to aggression remains of particular relevance. Many people experience relative deprivation in everyday life due to global inequalities [24], but presumably not every experience of deprivation leads to aggression. It is therefore particularly important to look at the processes that lead to the subjective experience of deprivation being perceived as particularly serious. In our study, both the reduction of positive affect and aggressive affect, i.e., anger, were linked to the subjective experience of deprivation. Albeit speculative, it is not implausible to assume that both affective states can lead people to a higher sensitivity of unfairness, that is, they pay more attention to differences between their own situation and that of the other player. This then could even widen the perceived gap between what they have and what they believe they deserve, which in turn decreases positive and increases aggressive affect. However, negative emotions such as anger can also reinforce hostile attributions [23]. This makes social inequalities appear even more unjust, which in turn intensifies the experience of affect. In order to weaken this mediation, an attempt could be made to keep positive affect more stable and not allow negative affect to increase so strongly. Here, for example, emotion regulation strategies could help to reduce the intensification of affective reactions. Future studies should investigate whether such emotion regulation strategies can reduce this recursive effect of perceived deprivation.

Theoretically, our results represent a challenge because most theoretical approaches to explaining the influence of relative deprivation on aggression tend to assume linear processes, also for methodological reasons. However, our results point to more dynamic recursive relationships, which, apart from appraisal theories, have previously been less addressed. Hence, methodologically and theoretically, more attention should be paid to the possibility of recursive relationships between affective states, RD, and aggressive behavior in the future.

### Limitations and avenues for future studies

Although the present findings allow a more nuanced understanding of the RD-aggression link by suggesting that affective experiences are associated to RD perceptions and, in turn, linked to aggression, our findings are based on a partly exploratory investigation and should thus be replicated by future research. Since the subjective perception of RD, but not the affective experience linked to RD, were manipulated, we cannot infer that the pathways assumed in the mediation model reflect the causal pathways of processes linking RD and affect to aggression [27]. Thus, further experimental research is needed to substantiate our study’s conclusions. Moreover, future studies should manipulate positive and aggressive affect to enhance the knowledge of which affective experience can alter the perception of RD. Similarly, future experiments could be designed to directly measure affective experiences during the game rather than retrospectively, which could show how these processes evolve over time.

## Conclusions

These limitations notwithstanding, our findings could potentially explain a large number of real-life observations, like the vicious cycle of anger due to increasing inequalities and leading to violent extremism, where angry and relatively deprived people seem to selectively perceive evidence in support of their subjective feeling of deprivation which make them in turn feel angrier. Given that many parts of the world are facing turbulent times because of increasing inequalities leading to aggression and violent extremism, understanding RD mechanism has become crucial for dealing with these major challenges. Clearly, more experimental research is needed for the identification of factors modulating aggression and for providing deeper insights into the mechanism linking or unlinking RD and aggressive behavior.

## Supplementary Information

Supplementary Material 1.

## Data Availability

The datasets used and/or analyzed during the current study are available from the corresponding author on reasonable request.

## Abbreviations

PPRD: Personal perception of relative deprivation
PSAP: Point Subtraction Aggression Paradigm
RD: Relative deprivation
