# Supplementary material for: The unfairness we feel: How positive and aggressive affect could shape relative deprivation and aggression
Source: BMC Psychol. 2025 Apr 17;13:392. doi: 10.1186/s40359-025-02732-x (PMC12004685; doi:10.1186/s40359-025-02732-x)
Supplement: Supplementary file 1 — Supplementary Material 1. [file 40359_2025_2732_MOESM1_ESM.docx]

Supplementary Material

The main analysis combined data from two experiments. Sixty-one (39 women, 10 men, 12 non-specified, Mage = 21.34, age range: 18 – 26 years) and 123 (36 women, 86 men, 1 non-specified, Mage = 22.49, age range: 18 – 31 years) undergraduate students participated in Experiment 1 and 2, respectively. Both experiments included an additional manipulation of group context. Before the RD game, participants first categorized themselves as belonging to the ingroup and the outgroup in terms of their study major for the Experiment 1 (i.e., “I belong to the group of psychology students”, “I belong to the group of business and administration students”), and in terms of their university in Experiment 2 (“I belong to the University of X”, “I belong to the University of Y”). Analogues to the results reported in the main manuscript, we analyzed the effects of the groups context manipulation in a 2 (RD-manipulation) x 2 (group context) ANCOVA with sample as covariate for the different dependent variables (see **Table S1**). Group context exerted no effect on gain differences, subjective RD, emotions, or aggressive behavior, all *p*s > .16. The total sample had a power of 1-*β* = .80 at *α* = .05 to detect an effect of *η^2^_p_* = .04 (small-to-medium by convention) of the group context manipulation. Further analysis, including group context, are reported below. Variable correlations can be found in **Table S2**.

**Social Dominance Orientation**

Participants in the experiments completed the Social Dominance Orientation (SDO) short version questionnaire (von Collani, 2002) at the start of the procedures. Using regression analysis, we explored whether SDO moderated aggressive behavior following the induction of RD, and if effects of Ingroup vs. Outgroup Context depended on a participant’s level of SDO. The internal consistency of the SDO questionnaire was acceptable, Cronbach’s α = .70. **Table S3** summarizes the regressions’ results for main effects only and the full factorial model (Outgroup and RD condition dummy coded, SDO scores z-standardized). Except for the tentative effect of RD condition, there were no other effects on aggressive behavior (i.e., the rate of destroyed coins per second in the point subtraction game).

**Disgust Propensity**

Because disgust has been shown to affect aggressive behavior (Pond et al., 2012), we also assessed participants’ moral- (5 items) and pathogen-disgust (6 items) on a five-point scale (Eickmeier et al., 2019) as a potential moderators following the induction of RD. The internal consistencies for the moral and pathogen disgust scales were acceptable, Cronbach’s α = .82 and .75. **Tables S4 and S5** summarize the regressions’ results (Outgroup and RD condition dummy coded, Moral and Pathogen Disgust scale scores z-standardized). Increased moral disgust was associated with reduced aggression in the main effects only model, but no effects related to moral disgust were obtained in the full factorial model. Conversely, increased pathogen disgust was linked to increased aggression in both the main effects only and the full factorial model, and pathogen disgust qualified the effect of the RD manipulation. Simple slope analysis showed that the RD condition led to higher levels of aggression at lower (M - 1SD) levels of pathogen disgust, *b* = 0.21, *SE* = 0.09, *p* = .02, but not at the mean or higher (M + 1SD) levels of pathogen disgust, *b* = 0.09, *SE* = 0.06, *p* = .17 and *b* = -0.04, *SE* = 0.08, *p* = .62, respectively. While the main effect suggests disgust could provoke aggression when unable to get out of a competitive situation, the interaction replicates Pond et al. (2012) who found that disgust propensity promotes behavioral avoidance and buffers against the effects of instigators of aggression.

**Table S1**

Results of group context comparisons (ANCOVA).

| **DV** | **Factor** | **SS** | **df** | **MS** | **F** | **p** | **η^2^_p_** |
| --- | --- | --- | --- | --- | --- | --- | --- |
| Reward Diff. | Intercept | 19763.25 | 1 | 19763.25 | 106.54 | <.001 | .37 |
|  | Cov. | 0.09 | 1 | 0.09 | 0.00 | .982 | .00 |
|  | RD | 267820.73 | 1 | 267820.73 | 1443.83 | <.001 | .89 |
|  | OG | 35.39 | 1 | 35.39 | 0.19 | .663 | .00 |
|  | RD*OG | 2.95 | 1 | 2.95 | 0.02 | .900 | .00 |
|  | Error | 33203.40 | 179 | 185.49 |  |  |  |
| PPRD | Intercept | 221.41 | 1 | 221.41 | 208.40 | <.001 | .54 |
|  | Cov. | 0.15 | 1 | 0.15 | 0.14 | .708 | .00 |
|  | RD | 269.05 | 1 | 269.05 | 253.24 | <.001 | .59 |
|  | OG | 2.07 | 1 | 2.07 | 1.94 | .165 | .01 |
|  | RD*OG | 0.39 | 1 | 0.39 | 0.37 | .543 | .00 |
|  | Error | 190.17 | 179 | 1.06 |  |  |  |
| Aff. Exp. | Intercept | 290.60 | 1 | 290.60 | 252.04 | <.001 | .59 |
|  | Cov. | 0.26 | 1 | 0.26 | 0.23 | .633 | .00 |
|  | RD | 3.93 | 1 | 3.93 | 3.40 | .067 | .02 |
|  | OG | 0.64 | 1 | 0.64 | 0.56 | .457 | .00 |
|  | RD*OG | 0.06 | 1 | 0.06 | 0.06 | .814 | .00 |
|  | Error | 206.39 | 179 | 1.15 |  |  |  |
|  | affect | 13.02 | 1 | 13.02 | 5.92 | .016 | .03 |
|  | affect*Cov. | 2.89 | 1 | 2.89 | 1.31 | .253 | .01 |
|  | affect*OG | 0.75 | 1 | 0.75 | 0.34 | .561 | .00 |
|  | affect*RD | 120.52 | 1 | 120.52 | 54.81 | <.001 | .23 |
|  | affect*RD*OG | 0.12 | 1 | 0.12 | 0.05 | .816 | .00 |
|  | Error (affect) | 393.58 | 179 | 2.20 |  |  |  |
| Aggression | Intercept | 3.52 | 1 | 3.52 | 38.82 | <.001 | .18 |
|  | Cov. | 1.34 | 1 | 1.34 | 14.72 | <.001 | .08 |
|  | RD | 0.37 | 1 | 0.37 | 4.02 | .046 | .02 |
|  | OG | 0.01 | 1 | 0.01 | 0.09 | .763 | .00 |
|  | RD*OG | 0.00 | 1 | 0.00 | 0.04 | .837 | .00 |
|  | Error | 16.24 | 179 | 0.09 |  |  |  |

*Note*. DV = dependent variable. SS = Square sums. df = degrees of freedom. MS = mean square. Cov. = Covariate (sample). RD = relative deprivation manipualtion. PPRD = personal perception of relative deprivation.

**Table S2**

Variables correlation matrix.

| **Variable** | **1** | **2** | **3** | **4** | **5** | **6** | **7** | **8** | **9** |
| --- | --- | --- | --- | --- | --- | --- | --- | --- | --- |
| 1. OG |  |  |  |  |  |  |  |  |  |
| 2. RD | -.01 |  |  |  |  |  |  |  |  |
| 3. SDO | .05 | -.08 |  |  |  |  |  |  |  |
| 4. PD | -.08 | .05 | -.04 |  |  |  |  |  |  |
| 5. MD | -.08 | .08 | -.02 | .06 |  |  |  |  |  |
| 6. Reward Diff. | .00 | .94** | -.05 | .01 | .07 |  |  |  |  |
| 7. PPRD | .06 | .76** | -.02 | -.06 | .05 | .74** |  |  |  |
| 8. Aggr. Aff. | -.01 | .31** | -.02 | .05 | .20** | .29** | .51** |  |  |
| 9. Pos. Aff. | .07 | -.53** | .08 | -.02 | -.10 | -.54** | -.64** | -.43** |  |
| 10. Aggression | .02 | .14 | -.01 | -.14 | .21** | .15* | .33** | .26** | -.15* |

*Note*. OG = Outgroup dummy variable, RD = relative deprivation dummy variable, SDO = Social Dominance Orientation scale mean, PD = Pathogen Disgust subscale mean, MD = Moral Disgust subscale mean, PPRD = personal perception of relative deprivation scale mean, Aggr. Aff. = aggressive affect scale mean, Pos. Aff. = positive affect scale mean. ** Correlation is significant at the 0.01 level (2-tailed). * Correlation is significant at the 0.05 level (2-tailed).

**Table S3**

Results from Linear Regression of Aggressive Behavior on Study Conditions and Social Dominance Orientation

| **Model** | **Predictor** | ***B*** | ***SE*** | ***β*** | ***t*** | ***p*** |
| --- | --- | --- | --- | --- | --- | --- |
| main effects | Intercept | 0.16 | 0.04 |  | 4.04 | <.001 |
|  | OG | 0.01 | 0.05 | .02 | 0.24 | .810 |
|  | RD | 0.09 | 0.05 | .14 | 1.91 | .058 |
|  | zSDO | 0.00 | 0.02 | .01 | 0.07 | .948 |
|  |  |  |  |  |  |  |
| full factorial | Intercept | 0.17 | 0.05 |  | 3.74 | <.001 |
|  | OG | 0.00 | 0.07 | .00 | 0.00 | .998 |
|  | RD | 0.07 | 0.06 | .12 | 1.13 | .260 |
|  | zSDO | 0.06 | 0.05 | .18 | 1.24 | .216 |
|  | OG × RD | 0.02 | 0.09 | .03 | 0.23 | .816 |
|  | OG × zSDO | -0.08 | 0.07 | -.16 | -1.12 | .265 |
|  | RD × zSDO | -0.08 | 0.06 | -.17 | -1.21 | .229 |
|  | OG × RD × zSDO | 0.08 | 0.10 | .12 | 0.85 | .396 |

*Note*. OG = Outgroup dummy variable, RD = relative deprivation dummy variable, zSDO = z-standardized Social Dominance Orientation scale mean.

**Table S4**

Results from Linear Regression of Aggressive Behavior on Study Conditions and Moral Disgust

| **Model** | **Predictor** | ***B*** | ***SE*** | ***β*** | ***t*** | ***p*** |
| --- | --- | --- | --- | --- | --- | --- |
| main effects | Intercept | 0.16 | 0.04 |  | 4.12 | <.001 |
|  | OG | 0.00 | 0.05 | .01 | 0.08 | .936 |
|  | RD | 0.09 | 0.05 | .15 | 2.02 | .045 |
|  | zMD | -0.05 | 0.02 | -.15 | -1.99 | .048 |
|  |  |  |  |  |  |  |
| full factorial | Intercept | 0.16 | 0.05 |  | 3.62 | <.001 |
|  | OG | 0.00 | 0.07 | .00 | -0.02 | .98 |
|  | RD | 0.08 | 0.06 | .14 | 1.32 | .19 |
|  | Outgroup × RD | 0.01 | 0.09 | .02 | 0.15 | .88 |
|  | zMD | -0.06 | 0.04 | -.19 | -1.34 | .18 |
|  | OG × zMD | 0.01 | 0.08 | .02 | 0.09 | .93 |
|  | RD × zMD | 0.03 | 0.06 | .08 | 0.50 | .62 |
|  | OG × RD × zMD | -0.03 | 0.10 | -.04 | -0.26 | .80 |

*Note*. OG = Outgroup dummy variable, RD = relative deprivation dummy variable, zMD = z-standardized Moral Disgust subscale mean.

**Table S5**

Results from Linear Regression of Aggressive Behaviour on Study Conditions and Pathogen Disgust

| **Study** | **Predictor** | ***B*** | ***SE*** | ***β*** | ***t*** | ***p*** |
| --- | --- | --- | --- | --- | --- | --- |
| main effects | Intercept | 0.16 | 0.04 |  | 4.14 | <.001 |
|  | OG | 0.02 | 0.05 | .03 | 0.47 | .642 |
|  | RD | 0.08 | 0.05 | .12 | 1.72 | .088 |
|  | zPD | 0.07 | 0.02 | .21 | 2.84 | .005 |
|  |  |  |  |  |  |  |
| full factorial | Intercept | 0.15 | 0.04 |  | 3.53 | <.001 |
|  | OG | 0.04 | 0.06 | .06 | 0.58 | .562 |
|  | RD | 0.09 | 0.06 | .14 | 1.39 | .167 |
|  | Outgroup × RD | -0.01 | 0.09 | -.02 | -0.12 | .903 |
|  | zPD | 0.15 | 0.05 | .49 | 3.18 | .002 |
|  | OG × zPD | -0.04 | 0.07 | -.09 | -0.60 | .551 |
|  | RD × zPD | -0.13 | 0.06 | -.30 | -2.05 | .042 |
|  | OG × RD × zPD | 0.01 | 0.09 | .01 | 0.10 | .920 |

*Note*. OG = Outgroup dummy variable, RD = relative deprivation dummy variable, zPD = z-standardized Pathogen Disgust subscale mean.
